# Supplementary material for: Macrophage-Secreted CSF1 Transmits a Calorie Restriction-Induced Self-Renewal Signal to Mammary Epithelial Stem Cells
Source: Cells. 2022 Sep 19;11(18):2923. doi: 10.3390/cells11182923 (PMC9496835; doi:10.3390/cells11182923)
Supplement: Supplementary file 1 [file cells-11-02923-s001.zip › Table S1.pdf]

Table S1. Antibodies and recombinant protein used in this study.

Antigen	Primary antibody	Manufacturer	Dilution	Application	Secondary antibody	Manufacturer	Dilution
CSF1	Mouse monoclonal	Santa Cruz Biotechnology Santa Cruz, CA	1:100	IHC<br><br>IF	Histofine, H1906-1<br><br>Cy3-conjugated donkey anti-mouse IgG	NICHIREI BIOSCIENCES INC. Tokyo, Japan<br><br>Jackson ImmunoResearch West Grove, PA	 <br>1:100
CSF1R	Mouse monoclonal	Santa Cruz Biotechnology	1:100	IHC<br><br>IF	Histofine, H1906-1<br><br>Cy3-conjugated donkey anti-mouse IgG	NICHIREI BIOSCIENCES INC.<br><br>Jackson ImmunoResearch	 <br>1:100
CD19	Rabbit polyclonal	GeneTex Hsinchu City, Taiwan, R.O.C	1:50	IF	Alexa Fluor 488-conjugated goat anti-rabbit IgG	Jackson ImmunoResearch	1:400
CD3	Mouse monoclonal	GeneTex	1:100	IF	Cy3-conjugated donkey anti-mouse IgG	Jackson ImmunoResearch	1:100
CD24	PE-conjugated, Rat monoclonal, clone M1/69	Biolegend San Diego, CA	1:33	Flow Cytometry	N/A	N/A	N/A
CD49	FITC-conjugated, Rat monoclonal, Clone GoH3	StemCell Technologies, Vancouver, Canada	1:33	Flow Cytometry	N/A	N/A	N/A
CD14	FITC-conjugated, Mouse monoclonal	Invitrogen Carlsbad, CA	1:150<br><br>1:100	Flow Cytometry<br><br>IF	N/A<br><br>Cy3-conjugated donkey anti-mouse IgG	N/A<br><br>Jackson ImmunoResearch	N/A<br><br>1:100
F4/80	Rabbit monoclonal	Cell signaling Inc. Danvers, MA	1:200<br><br>1:100	IHC<br><br>IF	Histofine, H1906-1<br><br>Alexa Fluor 488-conjugated goat anti-rabbit IgG	 <br>Jackson ImmunoResearch	 <br>1:400
Stat1	Rabbit monoclonal	Invitrogen	1:50	IF	Alexa Fluor 488-conjugated goat anti-rabbit IgG	Jackson ImmunoResearch	1:400
Stat6	Rabbit monoclonal	Invitrogen	1:50	IF	Alexa Fluor 488-conjugated goat anti-rabbit IgG	Jackson ImmunoResearch	1:400
M-CSFR Neutralizing antibody	Rat monoclonal, clone AFS98	GeneTex	0.1 mg /ml	Inhibition	N/A	N/A	N/A
M-CSF1 (Recombinant)		Biolegend	0.2 mg /ml	Expression	N/A	N/A	N/A

IHC – immunohistochemistry, IF – immunofluorescence, N/A – not applicable
