# Supplementary figures and images for: Macrophage-Secreted CSF1 Transmits a Calorie Restriction-Induced Self-Renewal Signal to Mammary Epithelial Stem Cells
Source: Cells. 2022 Sep 19;11(18):2923. doi: 10.3390/cells11182923 (PMC9496835; doi:10.3390/cells11182923)

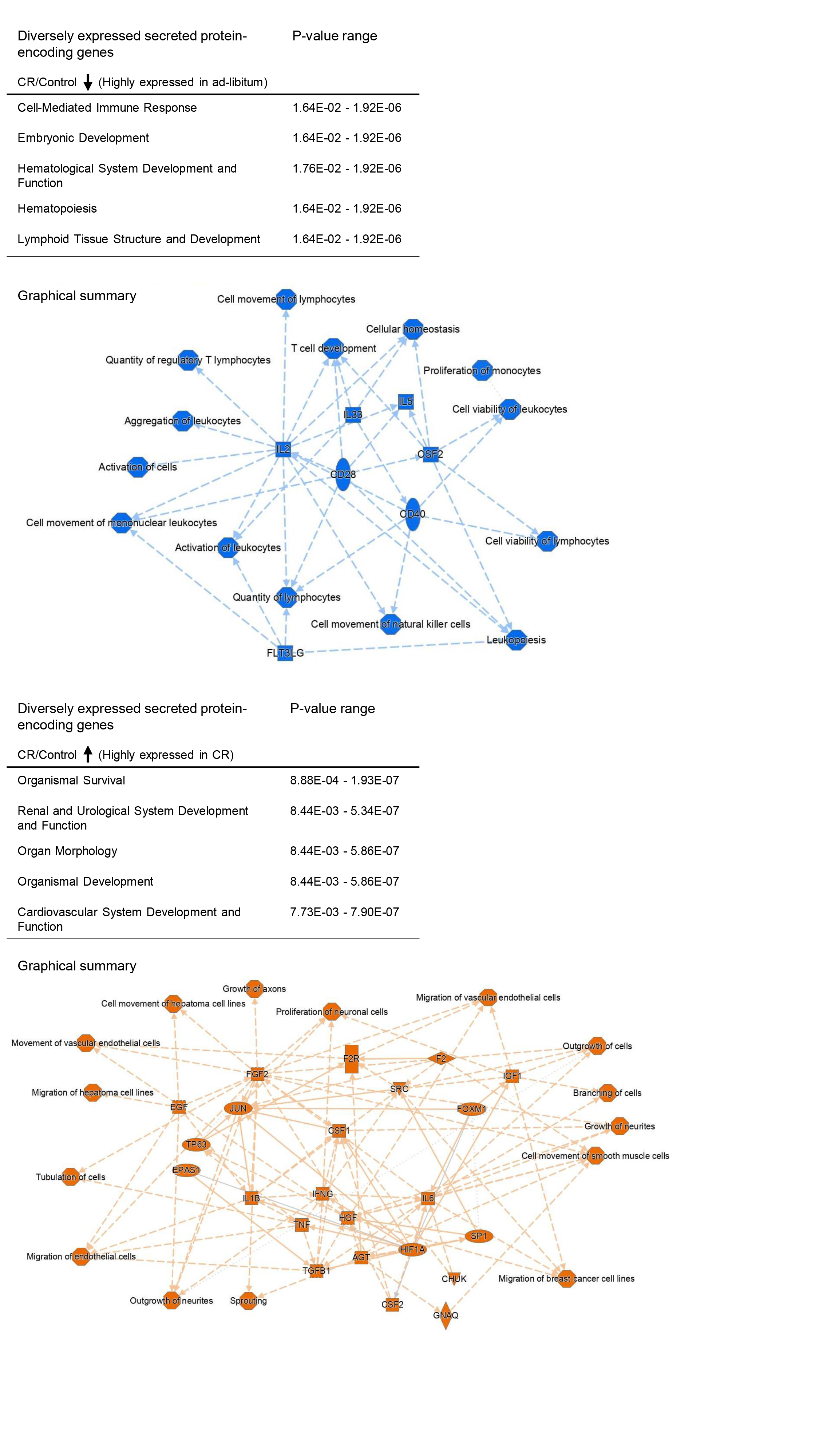

Supplement: Supplementary file 1 [file cells-11-02923-s001.zip › Figure S1.Tiff]

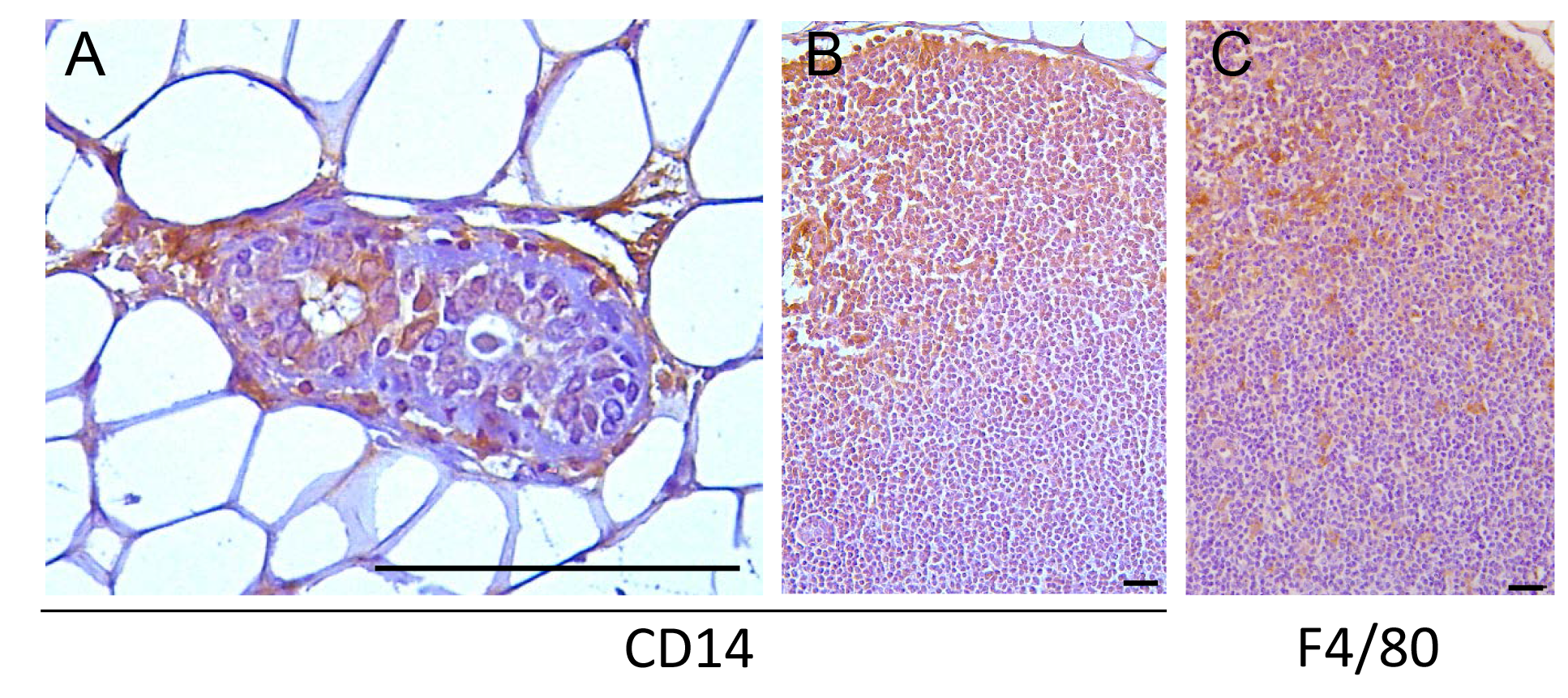

Supplement: Supplementary file 1 [file cells-11-02923-s001.zip › Figure S2.Tiff]
